# A new model to predict intravenous immunoglobin-resistant Kawasaki disease

**DOI:** 10.18632/oncotarget.21083

**Published:** 2017-09-19

**Authors:** Wang Hua, Yameng Sun, Ying Wang, Songling Fu, Wei Wang, Chunhong Xie, Yiying Zhang, Fangqi Gong

**Affiliations:** ^1^ Children's Hospital, Zhejiang University School of Medicine, Hangzhou 310052, PR China

**Keywords:** Kawasaki disease, IVIG resistant, independent risk factors, predictive model

## Abstract

**Objectives:**

To clarify the independent risk factors and construct predictive model for intravenous immunoglobin (IVIG)-resistant KD (IVIGRKD).

**Results:**

The ratio of male to female in the overall samples was 1.62:1 and the incidence of IVIGR was 17.9%. Multivariate regression analysis showed that the OR (95% CI) values of fever duration ≥ 7 days, delayed diagnosis, gamma-glutamyl transferase ≥ 25 U/L, serum sodium ≤ 135 mmol/L, neutrophil-to-lymphocyte ratio ≥ 2.8 and platelets ≤ 350 × 10^9^/L were 2.94 (2.17–4.00), 1.64 (1.07–2.53), 1.38 (1.07–1.79), 1.68 (1.30–2.19), 1.58 (1.22–2.06) and 1.39 (1.08–1.80), respectively. Based on these OR values, a new predictive model was established with an AUC of 0.685, a sensitivity of 60.7% and a specificity of 66.5%, and showed superiority to formerly reported models. Further analysis of patients ≤ 6 months old gave rise to improved predictions for IVIGRKD with an AUC of 0.746 relative the new model for the total samples.

**Materials and Methods:**

A total of 2,126 KD cases were enrolled in this study. Clinical indicators showing significant differences were screened using univariate analysis, and the independent risk factors were further elucidated using multivariate regression analysis. A new model was constructed, and the predictive ability was evaluated with the area under the curve (AUC) value and the sensitivity and specificity by using the receiver operating characteristic (ROC) curve.

**Conclusions:**

The new model for predicting IVIGRKD in this study is superior to those reported previously, and further analysis of patients with IVIGRKD younger than 6 months old allowed optimization of the predictive model.

## INTRODUCTION

Kawasaki disease (KD) is an acute systemic vasculitis of unknown etiology that predominantly affected coronary arteries. The disorder occurs worldwide and has replaced rheumatic fever as the number one cause of acquired heart disease in children. Intravenous immunoglobin (IVIG) resistance (IVIGR) put patients with KD at high risk for coronary artery lesions (CALs) and increased the treatment burden. Japan's 15th National Survey Data [1] showed that IVIGR was an independent risk factor for KD complicated by giant coronary aneurysms, with an OR (95%CI) of 43.6 (14.4–131). Numerous studies [2–7] have been conducted on predicting IVIG-resistant KD (IVIGRKD) and have been playing great guiding roles in early recognition and improve clinical outcomes at that time.

New problems have also arisen with an increased awareness of clinical features of IVIGRKD. Beijing Children's Hospital [5] reported 1,177 cases of KD from 2002 to 2010 for which the sensitivity and specificity of predicting IVIGRKD when using the Egami method were 21.4% and 86.6%, respectively, and when using the Kobayashi method were 48.8% and 71.6%, respectively. In 2008, Tremoulet et al. [6] in San Diego retrospectively analyzed 362 KD cases, and the sensitivity and specificity of predicting IVIGRKD using the Egami method were 38.3% and 83.85%, respectively; therefore, a new scoring method was developed with the sensitivity of 73.3% and the specificity of 61.9%. However, different prediction results were obtained for different ethnic groups, e.g., the sensitivities for Caucasian and Asian populations were 81.3% and 66.7%, respectively. These observations indicate that the previously reported scoring systems need to be updated, and different countries and ethnic groups will require different models to predict IVIGRKD.

In this study, a large sample retrospective study was performed to delineate the risk profiles for patients with IVIGRKD. Based on these data, a new predictive model was constructed, compared with those formerly reported, and optimized by age stratified.

## RESULTS

### Sample collection

Our hospital's inpatient data for six consecutive years from Jan. 2009-Dec. 2014 were collected from the database. A total of 2,126 cases were enrolled in this study, who were diagnosed with KD at the time of admission, and received initial IVIG administration at a dose of 2 g/kg.

### Comparison between IVIGRKD and IVIG-responsive KD patients

The ratio of male to female in the total sample was 1.60:1 and the incidence of IVIGR was 17.9%. Table 1 shows that fifteen indicators were significantly higher in the IVIG nonresponders than in the IVIG responders, including the gender ratio, total fever duration, days of hospitalization, the rate of delayed diagnosis, the CALs rate, erythrocyte sedimentation rate, C-reactive protein, white blood cell count, neutrophil ratio, absolute neutrophils, neutrophil-to-lymphocyte ratio, alanine aminotransferase, aspartate transaminase, total bilirubin, gamma-glutamyl transferase. Ten indicators were significantly lower in the IVIG nonresponders than in the IVIG responders, including days of illness at initial IVIG treatment, lymphocyte ratio, absolute lymphocytes, eosionphil ratio, absolute eosionphils, platelets, albumin, serum sodium, potassium and calcium.

**Table 1:** Clinical features of patients with IVIGRKD

Variables		Responsive (A)		Resistant (B)		U/X2	*P*
		*n*	median (p25-p75)/*n*(%)	*n*	median (p25-p75)/*n*(%)		
Male-to-female ratio		1746	1.52: 1	380	2.06: 1	6.57	0.01
Age	≤ 6 months, %	1746	275 (15.8)	380	43 (11.3)	4.965	0.084
	6 month-5years, %		1232 (70.6)		285 (75.0)	4.965	0.084
	> 5 years, %		239 (13.7)		52 (13.7)	4.965	0.084
illness days at admission, days		1746	6 (5–7)	380	6 (4–7)	–1.048	0.295
Number of major dignostic criteria		1746	4 (3–5)	380	4 (3–5)	–0.703	0.482
Days of illness at initial IVIG treatmetn, days		1746	6 (5–8)	380	6 (5–7)	–3.948	< 0.001
Total fever duration, days		1746	7 (6–8)	380	9 (7–10)	–11.204	< 0.001
Days of hospitalization, days		1746	6 (5–8)	380	9 (7–13)	–18.918	< 0.001
incomplete KD, %		1746	1025 (58.7)	380	214 (56.3)	0.733	0.212
Delayed diagnosis, %		1746	126 (7.2)	380	39 (10.3)	4.046	0.031
CALs, %		1735	370 (21.3)	380	118 (31.1)	16.617	< 0.001
ESR, mm/h		1584	64 (44–88)	360	73 (44–95)	–2.629	0.009
CRP, mg/L		1592	72 (34–122)	364	89 (45–157)	–4.526	< 0.001
WBC, × 10^9^/L		1608	12.4 (8.9–16.3)	364	13.4 (9.3–18.2)	–3.14	0.002
NE, %		1608	60.9 (46.1–73.3)	364	68.2 (51.9–82.3)	–6.56	< 0.001
Absolute NE, × 10^9^/L		1608	7.4 (4.3–11.1)	364	8.5 (5.5–13.4)	–5.008	< 0.001
LY, %		1608	28.5 (18.8–41.1)	364	21.2 (11.4–35.5)	–7.185	< 0.001
Absolute LY, × 10^9^/L		1608	3.3 (2.2–4.7)	364	2.7 (1.5–4.2)	–5.339	< 0.001
NLR,		1608	2.2 (1.1–3.8)	364	3.3 (1.5–7.1)	–7.001	< 0.001
EO, %		1604	2.3 (1.0–4.5)	363	1.8 (0.7–3.6)	–4.03	< 0.001
Absolute EO, × 10^9^/L		1604	0.28 (0.12–0.52)	363	0.23 (0.09–0.48)	–2.514	0.012
MO, %		1604	5.6 (3.4–8.2)	363	5.4 (2.9–8.1)	–1.414	0.157
Absolute MO, × 10^9^/L		1604	0.66 (0.40–1.01)	363	0.68 (0.39–1.09)	–0.824	0.41
Hemoglobin, g/L		1607	107 (100–114)	363	107 (99–113)	–1.101	0.271
Platelet, × 10^9^/L		1607	368 (291–460)	363	343 (258–445)	–3.28	0.001
ALT, U/L		1702	21 (12–46)	375	29 (14–78)	–4.96	< 0.001
AST, U/L		1702	30 (23–43)	375	34 (24–56)	–4.25	< 0.001
Albumin, g/L		1702	36.5 (33.7–39.1)	375	35.3 (31.3–38.4)	–5.017	< 0.001
TBil, μmol/L		1701	4.3 (2.9–6.5)	375	5.3 (3.4–10.3)	–5.962	< 0.001
GGT, U/L		1701	21 (10–68)	375	35 (13–102)	–4.749	< 0.001
Sodium, mmol/L		1590	137 (134–139)	348	135 (133–138)	–7.418	< 0.001
Chloride, mmol/L		1589	105 (103–108)	347	105 (102–107)	–0.998	0.318
Potassium, mmol/L		1590	3.7 (3.4–4.1)	348	3.6 (3.2–4.0)	–4.047	< 0.001
Calcium, mmol/L		1588	1.13 (1.07–1.19)	346	1.12 (1.06–1.18)	–2.121	0.034

IVIGRKD: intravenous immunoglobin–resistant Kawasaki Disease, CALs: coronary artery lesions, ESR: erythrocyte sedimentation rate, CRP: C-reactive protein, WBC: white blood cell count, NE: neutrophils, LY: lymphocytes, NLR: neutrophil-to-lymphocyte ratio, EO: eosionphils, MO: monocytes, ALT: alanine aminotransferase, AST: aspartate transaminase, TBil: total bilirubin, GGT: gamma-glutamyl transferase.

### Analysis of independent risk factors, and the establishment and optimization of the new predictive model for IVIGRKD

Table 2 showed that the independent risk factors for IVIGRKD were prolonged total fever duration, delayed diagnosis, higher neutrophil-to-lymphocyte ratio and gamma-glutamyl transferase, lower serum sodium and platelets. Further analyses showed that the OR (95%CI) values of fever duration ≥ 7 days, delayed diagnosis, gamma-glutamyl transferase ≥ 25 U/L, serum sodium ≤ 135 mmol/L, neutrophil-to-lymphocyte ratio ≥ 2.8 and platelets ≤ 350 × 10^9^/L were 2.94 (2.17–4.00), 1.64 (1.07–2.53), 1.38 (1.07–1.79), 1.68 (1.30–2.19), 1.58 (1.22–2.06) and 1.39 (1.08–1.80), respectively (Table 3). Based on these OR values, a new predictive model was established with an AUC of 0.685. For a cutoff point of 4, the sensitivity and specificity of IVIGRKD prediction were 60.7% and 66.5%, respectively (Table 4). Compared with previously reported IVIGR scoring systems, the new model developed in this study had a significantly higher AUC value than the Egami and Sano methods (0.685 ± 0.016 vs. 0.584 ± 0.018 and 0.578 ± 0.017, respectively; both *p* < 0.001) and tended to result in higher values relative to the Kobayashi method (0.685 ± 0.016 vs. 0.643 ± 0.018, *p* = 0.085).

**Table 2:** Multivariable logistic regression analysis of risk factors for IVIGRKD

Variables	B	S.E.	Wald	df	*p*	Exp(B)	95% CI for EXP(B)	
							Lower	Upper
Gender	–0.197	0.139	1.99	1	0.158	0.822	0.625	1.08
Total fever duration	0.3	0.032	85.053	1	< 0.001	1.349	1.266	1.438
Delayed diagnosis	1.394	0.352	15.655	1	< 0.001	4.032	2.021	8.043
ALT	0	0.001	0.023	1	0.88	1	0.997	1.002
AST	0	0.001	0.059	1	0.809	1	0.998	1.003
Albumin	–0.019	0.017	1.343	1	0.247	0.981	0.949	1.013
TBil	0.007	0.006	1.719	1	0.19	1.007	0.996	1.018
GGT	0.002	0.001	4.577	1	0.032	1.002	1	1.004
Sodium	–0.09	0.02	20.878	1	< 0.001	0.914	0.879	0.95
CRP	0	0.001	0.021	1	0.884	1	0.997	1.003
WBC	0.004	0.013	0.112	1	0.738	1.004	0.98	1.029
NLR	0.042	0.014	9.226	1	0.002	1.043	1.015	1.071
EO	–0.004	0.025	0.021	1	0.884	0.996	0.949	1.046
Platelet	–0.001	0.001	3.978	1	0.046	0.999	0.998	1

Hosmer and Lemeshow Test: *p* = 0.1, IVIGRKD: intravenous immunoglobin-resistant Kawasaki Disease, ALT: alanine aminotransferase, AST: aspartate transaminase, TBil: total bilirubin, GGT: gamma-glutamyl transferase, CRP: C-reactive protein, WBC: white blood cell count, NLR: neutrophil-to-lymphocyte ratio, EO: eosionphils.

**Table 3:** New scoring system to predict IVIGRKD

Variables	B	S.E.	Wald	df	p	Exp(B)	95% CI for EXP(B)		Score points
							Lower	Upper	
Total fever duration ≥ 7 days	1.079	0.156	47.674	1	< 0.001	2.942	2.166	3.997	2
Delayed diagnosis	0.497	0.219	5.131	1	0.024	1.644	1.069	2.527	1
GGT ≥ 25 U/L	0.325	0.13	6.304	1	0.012	1.384	1.074	1.785	1
Sodium ≤ 135 mmol/L	0.52	0.133	15.177	1	< 0.001	1.682	1.295	2.185	1
NLR ≥ 2.8	0.459	0.134	11.704	1	0.001	1.582	1.217	2.058	1
Platelet ≤ 350 × 10^9^/L	0.33	0.131	6.373	1	0.012	1.391	1.077	1.798	1
Total score									7

Hosmer and Lemeshow Test: *p* = 0.193, IVIGRKD: intravenous immunoglobin-resistant Kawasaki Disease, GGT: gamma-glutamyl transferase, NLR: neutrophil-to-lymphocyte ratio.

**Table 4:** Ability of scoring system to predict IVIGRKD

Area Under Curve	S.E.	95% CI for Area		Cutoff	Sensitivity	Specificity
		Lower	Upper			
0.685	0.016	0.652	0.717	3	0.828	0.401
				4	0.607	0.665
				5	0.405	0.847

IVIGRKD: intravenous immunoglobin-resistant Kawasaki Disease.

The independent risk factors for IVIGRKD in subgroup ≤ 6 months old were total fever duration, increased neutrophil-to-lymphocyte ratio and decreased serum sodium and platelets. Further analyses showed that the OR (95%CI) values of total fever duration ≥ 7 days, serum sodium ≤ 135 mmol/L, neutrophil-to-lymphocyte ratio ≥ 1.9 and platelets ≤ 350 × 10^9^/L were 3.63 (1.58–8.32), 2.02 (0.97–4.25), 3.11 (1.47–6.60) and 2.20 (1.02–4.74), respectively (Table 5). Based on these values, a predictive model was developed with an AUC of 0.746. For a cutoff point of 3, the sensitivity and specificity of predicting IVIGR in subgroup ≤ 6 months old were 82.1% and of 53.8%, respectively (Table 6).

**Table 5:** New scoring system to predict IVIGRKD ≤ 6 months old

Variables	B	S.E.	Wald	df	p	Exp(B)	95% CI for EXP(B)		Score points
							Lower	Upper	
Total fever duration ≥ 7days	1.289	0.424	9.249	1	0.002	3.628	1.581	8.323	2
Sodium ≤ 135 mmol/L	0.705	0.378	3.481	1	0.062	2.024	0.965	4.246	1
NLR ≥ 1.9	1.135	0.384	8.737	1	0.003	3.111	1.466	6.603	2
Platelet ≤ 350 × 10^9^/L	0.786	0.393	4.01	1	0.045	2.195	1.017	4.74	1
Total score									6

Hosmer and Lemeshow Test: p = 0.954, IVIGRKD: intravenous immunoglobin-resistant Kawasaki Disease, NLR: neutrophil-to-lymphocyte ratio.

**Table 6:** Ability of scoring system to predict IVIGRKD ≤ 6 months old

Area	S.E.	95% CI for Area		Cutoff	Sensitivity	Specificity
		Lower	Upper			
0.746	0.042	0.665	0.827	3	0.821	0.538
				4	0.59	0.752
				5	0.385	0.902

IVIGRKD: intravenous immunoglobin-resistant Kawasaki Disease.

The independent risk factors for IVIGRKD in subgroup > 5 years old were total fever duration and increased neutrophil-to-lymphocyte ratio. Further analyses showed that the OR (95%CI) values of total fever duration ≥ 9 days and 10 days were 4.39 (1.48–13.04) and 10.37 (4.15–25.92), respectively, and the OR values of neutrophil-to-lymphocyte ratio of 6–7, 7–8 and > 8 were 5.29 (1.70–16.47), 7.77 (1.70–35.54) and 9.13 (3.90–21.38), respectively.

## DISCUSSION

Data from the 20th National Survey of Japan [8] in 2007–2008 showed that 16.5% of KD patients received additional IVIG therapy. The 21st National Survey of Japan [9] data in 2009–2010 showed that IVIGRKD accounted for 16.6% of KD cases, which was similar to the rate of 17.9% that we found in this study.

IVIGR poses great harm to KD patients, and early identification and adoption of more aggressive treatment measures can effectively reduce vascular complications and improve prognoses. A cluster of risk factors for IVIGRKD have been reported in the past decade including elevated total bilirubin and aspartate transaminase [10], PCT > 0.5 ng/ml [11], band count > 10%, albumin ≤ 30 g/L and abnormal first echocardiographic results [12], elevated neutrophil ratio and alanine aminotransferase, low albumin (< 29 g/L) and pericardial effusion [13], elevated total bilirubin and C-reactive protein [14], gallbladder effusion and cholecystitis [15], C-reactive protein ≥ 10 mg/dL, lactate dehydrogenase ≥ 590 IU/L and hemoglobin ≥ 110 g/L [16]. Previous studies added to our understanding of IVIGRKD and also raised the demand on how to accurately predict its occurrence.

Clinical practice shows that the use of a combination of clinical parameters that are common, fast and easy to detect can result in stable predictions, thereby making it convenient to communicate among investigators and promote their worldwide application. In this study, univariate analysis was performed on the clinical parameters of all of the KD samples and of the samples of the IVIGRKD subgroup; we screened for the risk factors. Multivariate regression analysis was performed to determine the independent risk factors, and based on these results, models for predicting the development of IVIGR were constructed and compared with previous studies.

Kuo et al. [17] analyzed 185 cases of KD in 1999–2005 and found that decreased pre-treatment albumin (≤ 30 g/L) was associated with IVIGR, and Kim et al. [18] reported 285 cases of KD and found that C-reactive protein, aspartate transaminase, alanine aminotransferase and total bilirubin were elevated in the IVIGR group, similar to our findings. Han et al. [19] analyzed 185 cases of KD in 1995–1997 and found that days of illness at initial IVIG treatment was lower (5 days vs. 6 days), total fever duration (9 days vs. 6 days) was longer, and CAL was more frequent (76% vs. 43%) in the IVIGR group; these results were consistent with the results in the present study. Hwang et al. [20]. compared 23 cases of IVIGR and 206 cases of IVIG-responsive KD and found twenty-four hours after IVIG treatment, the sensitivities and specificities of the predictions for white blood cell count > 13.1 × 10^9^/L, neutrophil ratio > 51% and total protein < 72 g/L during IVIGR were (91%, 91% and 64%) and (89%, 76% and 78%), respectively; Because these indicators were measured after IVIG therapy, their implications for clinical predictions were inadequate; therefore, in this study, post-treatment measurements of laboratory indicators were not included in the predictive scoring system.

Multivariate analysis showed that the independent risk factors for IVIGRKD included long fever duration, delayed diagnosis, high gamma-glutamyl transferase, low blood sodium, increased neutrophil-to-lymphocyte ratio and platelets. A new model for IVIGR prediction were generated on the basis of the independent risk factors, with the AUC value of 0.685, the sensitivity of 60.7%, and the specificity of 66.5%. Predictive models for IVIGRKD being based on the indicators totally availabe in our data were also applicated to assess the present samples. The AUC value and sensitivity and specificity of IVIGR prediction on the data in this study were 0.584, 50.3% and 64.7%, respectively, when using the Egami method [2], were 0.578, 65.1% and 46.6%, respectively, when using the Sano method ^4^, and were 0.643, 0.53% and 0.693%, respectively, when using Kobayashi method [3]. These results indicate that the effectiveness of the new method was significantly better than either of the Egami or Sano methods and showed an increasing trend compared with the Kobayashi method. However, the new model was still not ideal with regard to sensitivity and specificity, indicating a need for further optimization.

KD occurs prevalently between 6 months and 5 years and clinical features of KD at the extremes of age are different from those at typical age [21, 22]. Multivariate regression analysis showed that the independent risk factors for IVIGRKD ≤ 6 months of age included prolonged total fever duration, elevated neutrophil-to-lymphocyte ratio, decreased platelets, and decreased sodium. Based on these results, the model for predicting younger IVIGRKD was generated, and the AUC value and sensitivity and specificity were 0.746, 59% and 75.2%, respectively, which indicating significant optimization compared to the values determined for the overall samples. Multivariate regression analysis showed that the independent risk factors for IVIGRKD > 5 years of age included prolonged total fever duration and increased neutrophil-to-lymphocyte ratio, and that the longer fever duration or the higher neutrophil-to-lymphocyte ratio, the greater risk for IVIGR. Because of only 2 risk factors scoring system was not established.

On the basis of our present data, more aggressive treatments should be taken including the second dose of IVIG, intravenous methylprednisolone or biologic medications, when children with KD have longer fever duration, delayed diagnosis, elevations of gamma-glutamyl transferase and neutrophil-to-lymphocyte ratio, and decreases of blood sodium and platelet. Furthermore, according to the new scoring system, all the variables are easy available, which makes physicians convenient to put it into clinical practice. Physicians have a deep understanding about treatments for KD that the higher scores it has, the higher possibility the additional treatments should be received.

In this study, a large retrospective sample data set from a single center was used, which resulted in some limitations. First, the signs and symptoms of KD were inspected and recorded by multiple clinical teams, and different clinicians had different levels of knowledge and experience of KD. Second, some items of the data were missing, which could have led to biases in statistical analyses; therefore, we have listed the number of cases for each group and each item. Because the data were collected over 6 consecutive years, the cases with large sample sizes were able to enhance the stability of the data, which compensates for the aforementioned limitations to a certain extent.

## MATERIALS AND METHODS

### Clinical data

Spreadsheets were created to collect the data of KD inpatients over six consecutive years between Jan. 2009 and Dec. 2014 in our hospital. The data included three categories: (1) epidemiological and clinical indicators including gender, age, illness days at admission, numbers of main diagnostic criteria at admission, days of illness at initial IVIG treatment, total fever duration, days of hospitalization, effectiveness of the IVIG therapy, echocardiographic results during hospitalization; (2) complete blood count indicators including erythrocyte sedimentation rate, C-reactive protein, white blood cell count, neutrophil ratio, lymphocyte ratio, neutrophil-to-lymphocyte ratio, eosionphil ratio, monocyte ratio, and the absolute counts of neutrophils, lymphocytes, eosionphils, and monocytes, hemoglobin, Platelets, measured before IVIG therapy; and (3) blood biochemical indicators (alanine aminotransferase, aspartate transaminase, albumin, total bilirubin and gamma-glutamyl transferase) and serum electrolytes (sodium, potassium, chloride and calcium), measured before IVIG therapy. This study was approved by the Research Ethics Committee of our hospital.

### Definitions

Complete KD was defined as an illness in a patient who met at least 5 of the 6 main symptoms [23], including fever, conjunctival hyperemia, lip and oral mucosal changes, polymorphous rash, changes in extremities, and cervical lymphadenopathy. Incomplete KD was defined as the presence of four or fewer of the main symptoms, regardless of CALs [24, 25], and other diseases that need to be distinguished from KD are excluded. Both complete and incomplete KD were enrolled in this study. The clinical features and laboratory indicators assisting the KD diagnosis include the presence of erythema at the Bacillus Calmette-Guérin (BCG) vaccination spot, perianal flushing and peeling, leukocytosis with neutrophils predominance, thrombocytosis, elevated C-reactive protein or erythrocyte sedimentation rate, anemia, hypoalbuminemia or hyponatremia, elevated alanine aminotransferase or gamma-glutamyl transferase, and urine white blood cell ≥ 10/high power field. IVIGRKD was defined as KD patients having a continued fever or recurrence of fever > 37.3°C at any time from 48 hours to two weeks after initial IVIG therapy accompanied by at least one of the main symptoms [5]. Delayed diagnosis of KD was defined as those who receive initial IVIG therapy 10 days after onset of KD [26]. Previously reported criteria [27] were used to diagnose CAL: (1) coronary artery diameter ≥ 2.5 mm in patients aged 0–3, ≥ 3.0 mm in patients aged 3–9 and ≥ 3.5 mm in patients aged 9–14, (2) the internal diameter of a segment's being at least 1.5-times as large as that of an adjacent segment, or (3) when the lumen was clearly irregular.

### Statistics

The median (P25-P75) was used to represent the measurement data, and the percentage of cases (%) was used to represent the count data. The nonparametric rank sum test was used for the comparison of the intergroup medians, whereas the Chi-square test was used for the comparison of the intergroup percentages. The indicators that showed differences in the univariate analysis were then subjected to multivariate logistic regression analysis, and the OR and 95% CI were calculated; *p* < 0.05 was considered to be statistically significant. Cutoff values were determined using the ROC curve, and the OR value was used to determine the score of an independent risk factor and construct a prediction model. The Hosmer-Lemeshow goodness of fit test was used to test the model, and when *p* was > 0.05, it indicated that the prediction model fit the sample data; the ROC curve was used to assess the area under the curve (AUC) and the sensitivity and specificity of the prediction model.

## Abbreviations

KD: kawasaki disease
IVIGR: intravenous immunoglobin resistance
CALs: coronary artery lesions
AUC: area under the curve
